# The research registry - answering the call to register every research study involving human participants

**DOI:** 10.1186/1745-6215-16-S2-P197

**Published:** 2015-11-16

**Authors:** Riaz Agha, Katharine Whitehurst, Harkiran K Sagoo

**Affiliations:** 1 Balliol College, Oxford, UK; 2 University College London, London, UK; 3 GKT School of Medicine, Kings College London, London, UK

## 

There is strong evidence that much research remains unpublished, especially studies with inconvenient or negative findings. Publication bias is skewing the research base to the detriment of scientific progress.

Current research registries suffer from bureaucracy, high author charges and a limited range study types that can be registered. The corollary is that less than 10% of observational studies are registered. This is despite the Declaration of Helsinki 2013 stating that: “Every research study involving human subjects must be registered in a publicly accessible database……Negative and inconclusive as well as positive results must be published or otherwise made publicly available.”

The Research Registry (http://www.researchregistry.com) is a ‘one-stop shop’ for registering all types of research studies as well as systematic reviews and meta-analyses. The data we collect is based on the WHO data set and includes some additional items. The aim is to adapt this resource to the needs of the users. The Research Registry will register research prospectively (as is best practice), and also retrospectively. It will record negative studies and ones where the outcome was suboptimal. It will thus provide a comprehensive scientific and historical record. It is open access, searchable, simple to use and free to register.

The Registry has recently been endorsed by the IDEAL Collaboration, an initiative to improve the quality of surgical research. We call on readers, authors, reviewers, editors and the scholarly community at large to encourage use of this service for the benefit of us all and future generations.

